# From Chilean Patagonia to Galapagos, Ecuador: novel insights on blue whale migratory pathways along the Eastern South Pacific

**DOI:** 10.7717/peerj.4695

**Published:** 2018-04-30

**Authors:** Rodrigo Hucke-Gaete, Luis Bedriñana-Romano, Francisco A. Viddi, Jorge E. Ruiz, Juan Pablo Torres-Florez, Alexandre N. Zerbini

**Affiliations:** 1 Instituto de Ciencias Marinas y Limnológicas, Universidad Austral de Chile, Valdivia, Chile; 2 Centro Ballena Azul, Valdivia, Chile; 3 Departamento de Genética e Evolucão, Universidade Federal de São Carlos, São Carlos, São Paulo, Brazil; 4 Marine Mammal Laboratory, Alaska Fisheries Science Center, NOAA, Seattle, Washington, D.C., USA; 5 Cascadia Research Collective, Olympia, Washington, D.C., USA; 6 Instituto Aqualie, Juiz de Fora, Minas Gerais, Brazil

**Keywords:** *Balaenoptera musculus*, Habitat use, International management, Migration, Satellite telemetry, Chiloense Ecoregion, State space models, Movement ecology, Marine conservation

## Abstract

**Background:**

The most traditional scheme for migration among baleen whales comprises yearly migrations between productive waters at high latitude summer feeding grounds and warmer waters at lower latitudes where whales calve and mate, but rarely feed. Evidence indicates, however, that large departures from this scheme exist among populations and individuals. Furthermore, for some populations there is virtually no information on migratory pathways and destinations. Such is the case of Chilean blue whales throughout the Eastern South Pacific; hence, the goal of this study was to assess its migratory behavior.

**Methods:**

Dedicated marine surveys and satellite tagging efforts were undertaken during the austral summer and early autumn on blue whale feeding grounds off Chilean Northern Patagonia (CNP) during 2013, 2015 and 2016. Positional data derived from satellite tags regarding movement patterns and behavior were analyzed using Bayesian switching first-difference correlated random walk models.

**Results:**

We instrumented 10 CNP blue whales with satellite transmitters and documented individual variation in departure time, northbound migratory routes and potential wintering grounds. The onset of migration occurred from mid/late austral autumn to well into the austral winter. Blue whales moved in various directions, but ultimately converged toward a general NW movement direction along a wide corridor exceeding 2,000 km. Area-Restricted Search behavior was exhibited within fjords and channels of CNP and also South of Galapagos Archipelago (GA) and northern Peru, but never during migration. Interestingly, dive profiles for one whale that reached GA showed a sharp and consistent increase in depth north of 5°S and extreme deep dives of up to 330 m.

**Discussion:**

Information derived from satellite tagged blue whales in this study is the first of its kind off the Eastern Southern Pacific. Our results provide valuable information on their migratory timing, routes and behavior on their northbound migration, particularly regarding the varied migratory plasticity for this particular population. Our results also highlight the first record of two complete migratory paths between CNP and GA and strengthen the hypothesis that GA waters correspond to a potential wintering destination for CNP blue whales. We further hypothesize that this area might be selected because of its biological productivity, which could provide feeding opportunities during the breeding season. Our results suggest that special efforts should be put forward to identify blue whale critical areas and understand key behavioral aspects in order to provide the basis for their conservation on a regional context (i.e., reducing potential ship strike and promote Marine Protected Area (MPA) implementation in Chile, Ecuador and Peru). Indeed, we suggest joint blue whale conservation efforts at the regional level in order to identify and determine potential threats and impacts and, most importantly, implement prospective management actions.

## Introduction

The most traditional conception of migration among baleen whales (Mysticeti) comprises a cyclic long-distance movement between summer-feeding in productive high latitude waters and over-wintering in warmer waters at lower latitudes, where they calve and mate, but rarely feed (Mackintosh & Wheeler, 1929; Mackintosh, 1942, 1965; Small, 1971; Bowen & Siniff, 1999). While several aspects of this pattern have been investigated for some populations (Mackintosh & Wheeler, 1929; Mackintosh, 1965; Geijer, Notarbartolo Di Sciara & Panigada, 2016), many authors have also provided insights into departures from the purported pattern, highlighting the relevance of further dissecting the large variation in migratory patterns.

There is evidence of individual whales suspending migration during episodes of vast food availability (Best, Sekiguchi & Findlay, 1995; Stamation et al., 2007; Barendse et al., 2010), observations of individuals skipping migration by remaining at breeding or feeding grounds throughout the year (Ingebrigtsen, 1929; Papastavrou & Van Waerebeek, 1998; Donovan, 1984; Palacios, 1999; Sirovic et al., 2004) and inter-oceanic exchange of migrating individuals (Pomilla & Rosenbaum, 2006; Stevick et al., 2010, 2014). Even though these exceptions might be related to the inherent plasticity of migratory patterns within a population (Craig & Herman, 1997), other migratory strategies are consistently different at the species level, such as in the bowhead whales (*Balaena mysticetus*) which never depart from the ice-edge (Ferguson et al., 2010) or Bryde’s whales (*Balaenoptera edeni/brydei*) which remain in tropical and temperate waters throughout the year (Kato & Perrin, 2008).

Blue whales (*Balaenoptera musculus*), are thought to fit their migratory routes and destinations to highly productive areas where dynamic oceanographic processes such as upwelling and frontal meandering occur and feeding expectations are fulfilled throughout the year (Branch et al., 2007b). This might be due to the enormous energetic requirements of these species (Goldbogen et al., 2011, 2015) that would prevent them from fasting for long periods. However, differences exist between populations of this species. In the Southern Hemisphere, a localized aggregation of pygmy blue whales (*B. m. brevicauda*) occurs in coastal waters off southern Australia during summer and autumn to feed on coastal krill (*Nyctiphanes australis*). This krill species appears to aggregate in response to enhanced, predictable productivity resulting from the summer–autumn wind-forced Bonney Coast upwelling along the continental shelf (Gill, 2002). Satellite tagged whales of this population have provided evidence of migration to potential breeding grounds off Indonesia. In this region, feeding might also occur by taking advantage of enhanced upwelling during the southeast monsoon season, which coincides with the presence of blue whales (Double et al., 2014). Within the Northern Indian Ocean, another population of blue whales appears to be resident, or at least performing small scale north-east migrations between Sri Lanka and the Arabian Sea, matching fluctuations in productivity triggered by the interplay of northeast and southwest monsoons (Stafford et al., 2011; Anderson et al., 2012; Randage et al., 2014). In the Eastern North Atlantic, feeding has been proposed to occur in mid-latitude waters off the Azores where blue whales spend prolonged periods of time until resuming their spring migration toward northern feeding grounds (Visser et al., 2011; Silva et al., 2013). While these studies provide evidence of large variability in blue whale migratory patterns, the seasonality, routes used, individual variability and the location of wintering destinations remain virtually unknown for some populations.

The Eastern Southern Pacific (ESP) population of blue whales is one of the least studied in the world. Early studies believed that ESP whales were Antarctic blue whales (*B. m. intermedia*), migrating to and from the Southern Ocean in a regular fashion (Kellogg, 1929; Brown, 1962; Mackintosh, 1965). However, the discovery of a mid-latitude feeding and nursing ground in Chilean Northern Patagonia (CNP, Hucke-Gaete et al., 2004) led to the development of several studies that suggested, in fact, that these ESP blue whales correspond to a separate population (originally suggested by Ramírez, 1983), possibly a new subspecies, the Chilean blue whale, with distinct distribution, migratory, genetic and acoustic patterns (Hucke-Gaete, 2004; Branch et al., 2007a, 2007b; Galletti et al., 2012; Buchan, Stafford & Hucke-Gaete, 2015; Torres-Florez et al., 2014, 2015; LeDuc et al., 2017). Recently, a plausible migratory link between CNP and Galapagos Archipelago (GA) was suggested from a single female blue whale photo-identified and genetically matched 10 years apart (Torres-Florez et al., 2015). This link was subsequently supported by other genetic and acoustic studies (Buchan, Stafford & Hucke-Gaete, 2015; Torres-Florez et al., 2014, 2015), however, this potential migratory path has never been recorded.

Using georeferenced data from individual ESP blue whales instrumented with satellite tags, we address the predictions of the hypothesis that blue whales feeding in CNP use GA as a wintering ground. Specifically, this work provides insights into previously unknown migratory pathways between summer and winter grounds, departure times, inter-individual variability and anecdotal information on diving patterns along the migratory pathways and the wintering destination.

## Methods

### Satellite tagging

A 17 m yacht (L/M Noctiluca) was used as the main searching platform while towing the tagging platform, a 7.6 m rigid-hulled inflatable boat (R/V Musculus) equipped with a bow platform and a 150 hp outboard engine. Satellite tags were deployed on 10 blue whales during the austral summer and early autumn at their feeding grounds off the Chilean Northern Patagonia (CNP) under permit #2267 to Rodrigo Hucke-Gaete (RHG) by the Subsecretaría de Pesca y Acuicultura, Ministry of Economy and Tourism, Chile. Whales were tagged in waters of the Chiloe inner sea during late March and early April in 2013 (*n* = 1), 2015 (*n* = 6) and 2016 (*n* = 3). Tags were deployed using a custom-modified compressed-air line thrower (ARTS/RN, Restech Norway, Heide-Jørgensen et al., 2001) set at pressures ranging between 10 and 14 bar. Two types of custom-designed transdermal implantable satellite tags were used: location-only (models SPOT5 (*n* = 3) and SPOT6 (*n* = 3)) and Argos-linked archival (MK10 (*n* = 4)) tags manufactured by Wildlife Computers (Redmond, Washington, D.C., USA). Both types of tags were equivalent regarding satellite-derived location recording, but differed mostly in the capability of gathering temperature and depth data by MK10. The tags were attached anterior to the dorsal fin of the whales and were equipped with an anchoring system comprised of two actively deployed flaps and a skirt of 10 backward-facing petals (a modified version of the anchors described in Gales et al., 2009). Tags deployed in 2013 and 2015 were programmed to transmit on a daily basis from March to May, every hour of the day (provided satellite coverage was available) up to a maximum of 500 messages per day. Starting in June, tags maintained their programming, except that transmissions occurred every other day. Tags from 2016 were programmed similarly to the other tags but the restriction of transmitting every other day after May was not implemented.

### State-space modeling

Raw Argos data was used, with the exception of a few remarkably extreme or biologically implausible locations that were removed in order to improve fitting (<4% of the total raw data). Data from whale #2 were split into two different tracks which were analyzed independently because of a gap in transmissions lasting approximately one month. Therefore, hereafter we will refer to the first part of this track (before the gap) and the second part (after the gap) as #2a and #2b respectively.

A Bayesian switching first-difference correlated random walk (DCRWS) model (Jonsen, Flemming & Myers, 2005) was fit to the raw Argos data. As this approach has been extensively described elsewhere (Jonsen, Flemming & Myers, 2005; Jonsen, Myers & James, 2007; Jonsen, 2016; Patterson et al., 2008) we will only briefly describe the procedure. ARGOS satellite tags provide location information with error in space and at irregular time intervals, thus DCRWS couple two stochastic models: a process model and an observation model. The process model predicts the future state of an animal given its current state. As animals are expected to switch their behavior along their paths, the process model to describe movement dynamics allows movement parameters to change between two discrete behavioral states by including a process model for each one (Morales et al., 2004). Metrics used for the process model were, mean turning angle, with a descriptive parameter θ_*b*_ distributed according to a beta distribution and, autocorrelation between speed and direction, with a descriptive parameter γ_*b*_ distributed according to a gamma distribution. Subscript “*b*” indicates the two possible behavioral states, assuming descriptive parameters differ between both behavioral modes. For each Markov Chain Monte Carlo (MCMC) sample, the behavioral modes assume a value of 1 or 2 yielding a model output that correspond to the average of the *n* MCMC re-samples, which then results into a continuous variable; higher values represent higher turning angle and speed/direction variability. Modes are then classified (conservatively) as follows: behavioral mode 1 (1–1.25) assumes a low turning angle and speed/direction variability and is classified as transit behavior, which in this case is associated to migratory behavior. Behavioral mode 2 (1.75–2) corresponds to higher turning angles and speed/direction variability, and is classified as area-restricted search (ARS), which might be associated with foraging activities. Unclassified behavior mode values fall between 1.25 and 1.75. State variables related to true locations and behavioral states were estimated at the individual level but assuming individuals share the same movement parameters using a joint estimation multi-level structure variant of the previously described model (Silva et al., 2013; Jonsen, 2016). Assuming identical movement parameters, although more simplistic, allows better state estimation in error-prone data sets like those derived from ARGOS, ultimately enhancing the capability of identifying individual differences in where and when whales show each behavioral state (Jonsen, 2016). Priors set for model parameters were modified according to other studies of satellite-tagged marine mammals (Jonsen, 2016).

The observation model relates the unobserved true locations predicted by the process model to the observed data (locations obtained from Argos). As the model assumes each location is observed at regular time-steps, all irregularly observed data within an assigned regular time-step are pulled to estimate the respective regular location. Error from each of these observations is accounted by using a *t*-distribution with different parameters depending on the corresponding ARGOS location class (Vincent et al., 2002; Jonsen, Flemming & Myers, 2005). Since migratory movements and wintering destinations of blue whales along the ESP had never been previously described, and we expected to receive transmissions at least until whales reached their wintering destination, the programmed duty-cycle used in most tags (explained in the section above) to ensure this led to the existence of gaps that prevented us from using a regular time-step of less than 48 h (Breed et al., 2011). According to this limitation, we fitted a version of the model (model 1) using this time-step for the entire data set with the objective of identifying general migratory patterns. However, for a subset of the data that comprised mostly information on whale movements at the beginning of the tracking time (mainly at CNP) and the entire data available for whale #10 (Table 1) we fitted a second identical model but with a time-step of 6 h (model 2) which permitted more detailed inspection of movement patterns at CNP and GA.

**Table 1:** Summary of satellite tag deployment data and movement descriptors of blue whales instrumented in Chilean Northern Patagonia.

ID	TAG Type	Transmission start	Transmission end	Track duration (days)	Distance travelled (km)	ARS Speed (mean ± SD) (km h^−1^)	Transit Speed (km h^−1^)
1	Spot	2013-04-01	2013-05-15	45	1028.6	0.97 ± 1.2 (0.05–5.7)	na
2a	Spot	2015-04-09	2015-04-25	17	509.7	1.14 ± 1.3 (0.1–3.8)	na
2b	Spot	2015-05-31	2015-07-06	37	313.2	0.36 ± 0.3 (0.02–0.9)	na
3	Spot	2015-04-14	2015-07-05	83	7086.1	na	4.17 ± 1.1 (2.0–6.1)
4	MK10	2015-04-09	2015-06-12	65	5102.9	na	3.55 ± 1.2 (0.8–7.1)
5	MK10	2015-04-16	2015-06-09	55	3923.7	na	4.05 ± 1.1 (2.0–6.3)
6	MK10	2015-04-17	2015-09-24	161	7420.3	0.52 ± 0.5 (0.01–2.4)	5.43 ± 1.7 (2.6–10.2)
7	MK10	2015-04-13	2015-05-03	21	143.7	0.34 ± 0.4 (0.02–1.1)	na
8	Spot	2016-04-10	2016-05-04	25	188.8	0.34 ± 0.3 (0.04–0.8)	na
9	Spot	2016-04-04	2016-04-25	23	648.0	na	3.1 ± 1.1 (1.9–4.0)
10	Spot	2016-04-05	2016-10-22	201	8820.1	0.78 ± 0.9 (0.03–3.9)	5.2 ± 1.1 (2.3–6.9)
					Pooled	0.67 ± 0.8 (0.01–5.7)	4.3 ± 1.4 (0.8–10.2)

Both models were fit in R (R Core Team, 2014) and JAGS (Plummer, 2003) using MCMC estimation methods. Two chains were run in parallel, producing a total of 120,000 MCMC samples each. The first 100,000 samples were discarded as burn-in, and one out of every 20 remaining samples was retained, for a total of 2,000 samples to form the posterior distribution of model parameter estimates. Model convergence was assessed by ascertaining whether posterior samples were stationary, the individual MCMC chains were well-mixed, within-chain sample autocorrelation was relatively low, and the Brooks–Gelman–Rubin potential scale reduction factors were ≤1.1 (Gelman & Rubin, 1992; Brooks & Gelman, 1998).

### Dive analysis

Dive data from MK10 tags were analyzed with the R package diveMove 1.4.0 (Luque, 2007). Depth measurements were recorded using the “time series” option available for programing MK10 tags. Sampling resolution for depth data was 75 s. Dive profiles were irregularly recorded in time as bouts (continuous recordings of time and depth every 75 s), with a minimum duration of 1 h per bout and a maximum of 4 h. As instrument resolution provides coarse measurements of the diving characteristics, we defined true dives as those that exceeded half-a body length (∼10 m) in depth in order to disregard surface breathing activity. After this correction the following basic dive statistics were calculated: (i) dive depth, defined as the maximum depth in meters that a whale reached during a single dive; (ii) dive duration, defined as the time elapsed between the start of a dive (exceeding 10 m) and the return to the surface (above 10 m); and (iii) inter-diving interval, defined as the time in seconds between the end of one dive and the start of the next one in one bout. These metrics were grouped accordingly with the regular time-steps designated for the State-Space model and calculated mean and SD values for each one of this bins when data was available. As light hours are expected to affect diving behavior of krill-tracking whales, we divided each bin in daytime and nighttime and calculated a separate mean and SD for each one.

## Results

**Figure 1:**
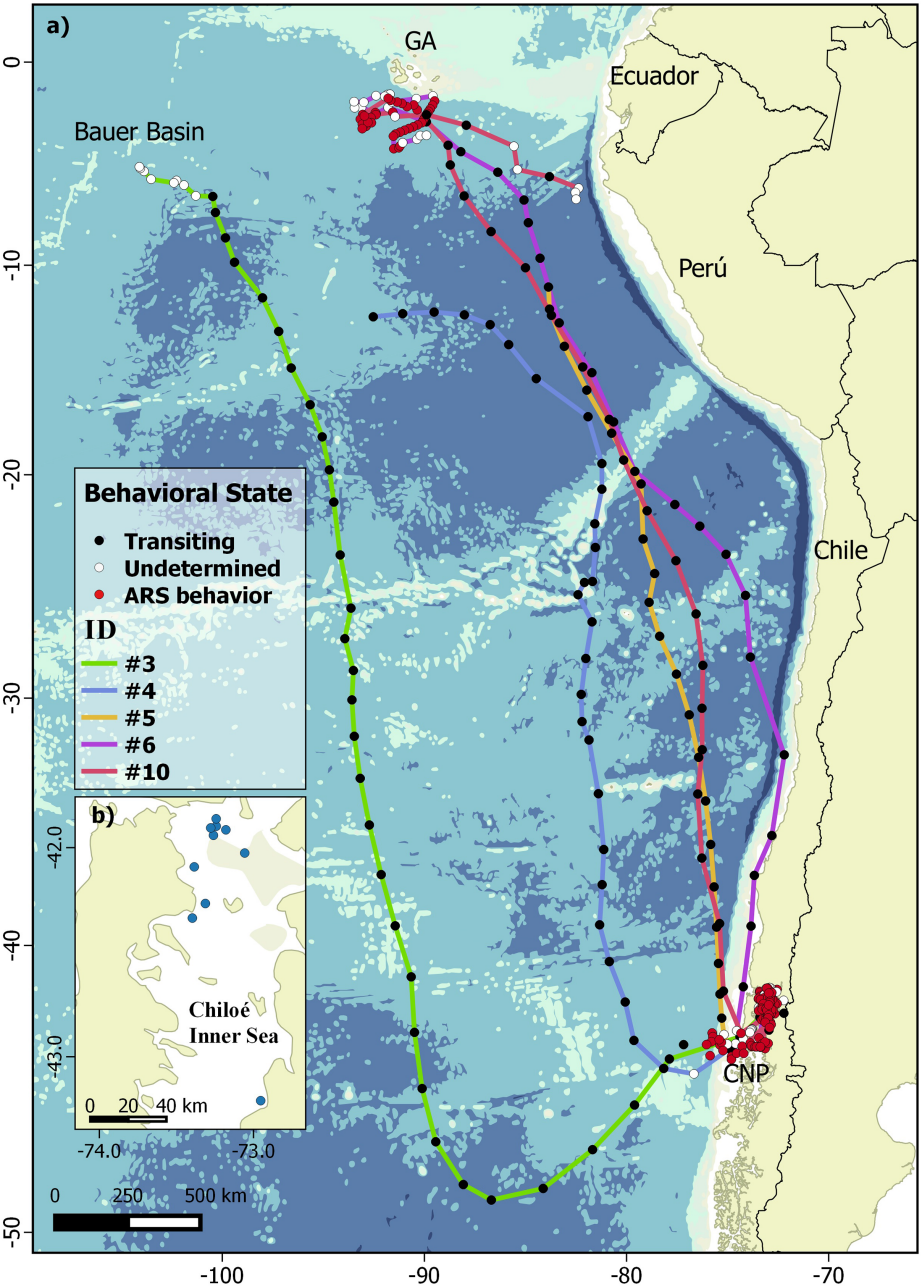
Bayesian switching first-difference correlated random walk (DCRWS) model-derived tracks of five wide-ranging blue whales tagged in Chilean Northern Patagonia. (A) DCRWS model-derived tracks of five wide-ranging blue whales tagged in Chilean Northern Patagonia’s feeding ground and migratory routes along the Eastern South Pacific, showing different behavioral states using model 1 (48 h time-step); (B) Tagging locations in the Chiloe inner sea.

As summarized in Table 1, seven out of 10 tags provided detailed information (i.e., including behavioral states) on how blue whales move and potentially behave at their feeding ground in CNP during the austral autumn and winter of 2013, 2015 and 2016. Of these, five provided valuable information on the migratory timings, routes and behavior on their northbound migration (Fig. 1). Effective transmission of all tags lasted on average 60.5 days (range = 17−201 days).

**Figure 2:**
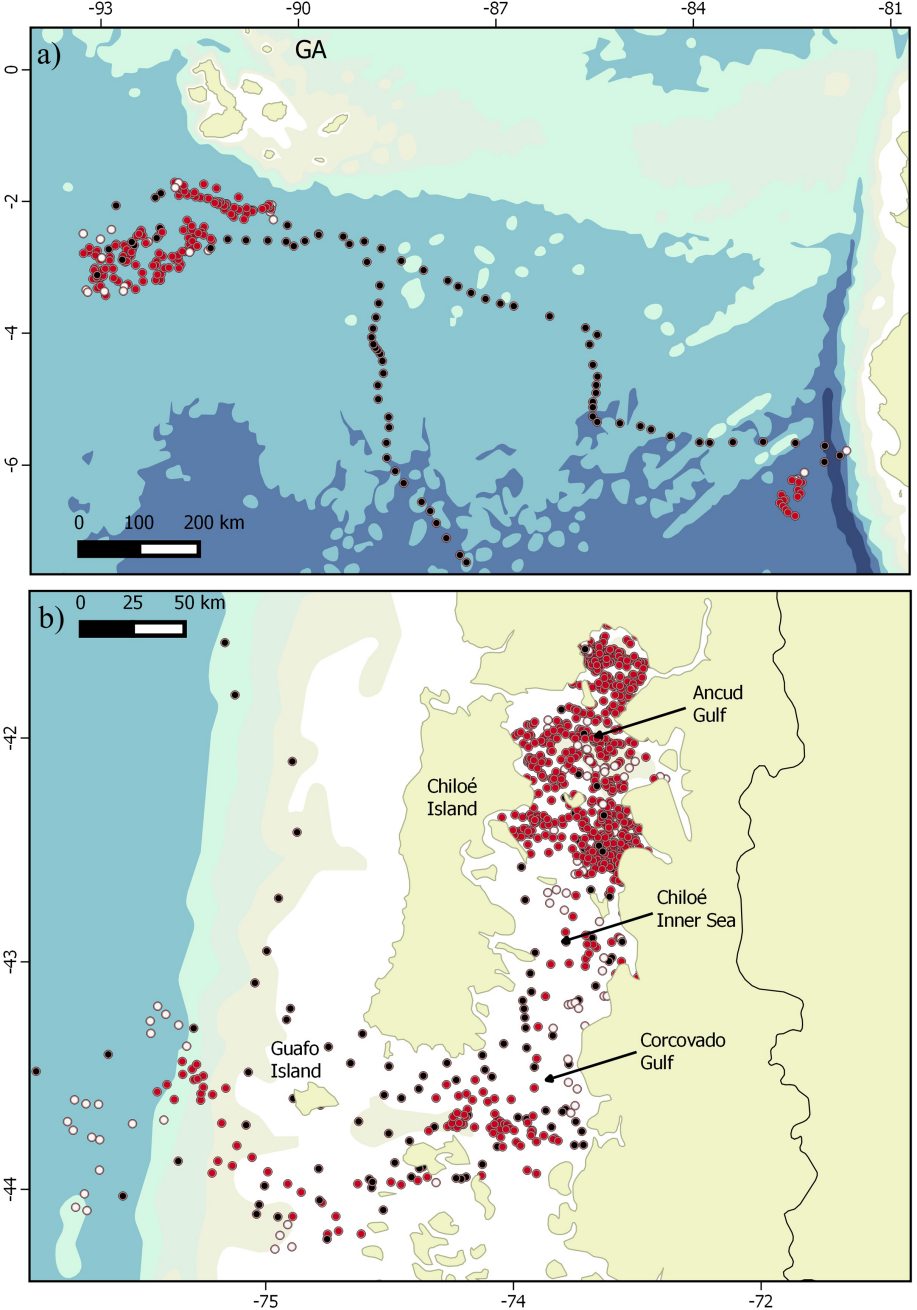
Transit and ARS behaviors shown by blue whales. Fine scale locations of behavioral states shown by blue whales. (A) Zoomed view of GA where ARS behavioral mode (red dots) was observed; black dots indicate transit and white dots undetermined behavior. (B) View of the CNP where all whales are grouped using model 2 (sixth time-step). Note that (A) and (B) represent different spatial scales.

Whales #1, #2, #7 and #8 only exhibited behavioral states (besides undetermined behavior) identified as ARS (Table 1) and were restricted to waters adjacent to the Chiloe Archipelago (Fig. 2). From these whales, those tagged in 2015 (#2a, #2b, #7, #8) remained within Ancud Gulf in an area of roughly 3,200 km^2^, while the one whale tagged in 2013 (#1) and one of the whales tagged in 2016 (#10) covered a larger area and performed an off-shore endeavor west of Guafo Island, reaching waters of more than 2,000 m depth and then returning to Chiloe’s Inner Sea (Fig. 2).

Whales #3, #4 and #5 only exhibited transit mode (Table 1) starting their migration soon after tag deployment. Whales #3, #4, #5, #6 and #10 presented distinct northward routes along ESP, with one whale (#3) migrating as far as 2,000 km west/southwest off the South American coast before heading north and reaching Bauer Basin during early July, some 1,500 km west/southwest off Isabela Island in GA (or ca. 2,500 km off mainland Ecuador) (Fig. 1). Whale #6, spent 44 days within the Ancud Gulf after tagging, then migrated along the coast of Chile changing its course northwest ca. 30°S and finally reaching waters South of GA after 40 days, where it stayed for at least 72 more days until transmission ceased. Whale #10, spent 104 days within the Chiloe inner sea in ARS, started the migration in late July, and moved directly to the GA where it arrived after 42 days. Subsequently, this individual spent ca. 39 days roaming extensively over an area of ca. 130,000 km^2^, and then moved SE toward Northern Peru where it remained in ARS and undetermined behavior for a further 16 days until transmissions ceased (Fig. 2). Among all whales, ARS behavior was detected at both CNP and GA. Irrespective of the model used (Fig. 1), migration was never interrupted by an eventual feeding opportunity. Although both models showed fair agreement with the results, model 2 allowed differentiating distinct areas where ARS behavior was performed within both CNP and GA. For instance, whale #10 showed clear ARS behavior in northern Peru, and allowed distinguishing between transitions (e.g., from ARS to transit and back to ARS) only using the high resolution model (model 2) (Figs. 1 and 2).

**Figure 3:**
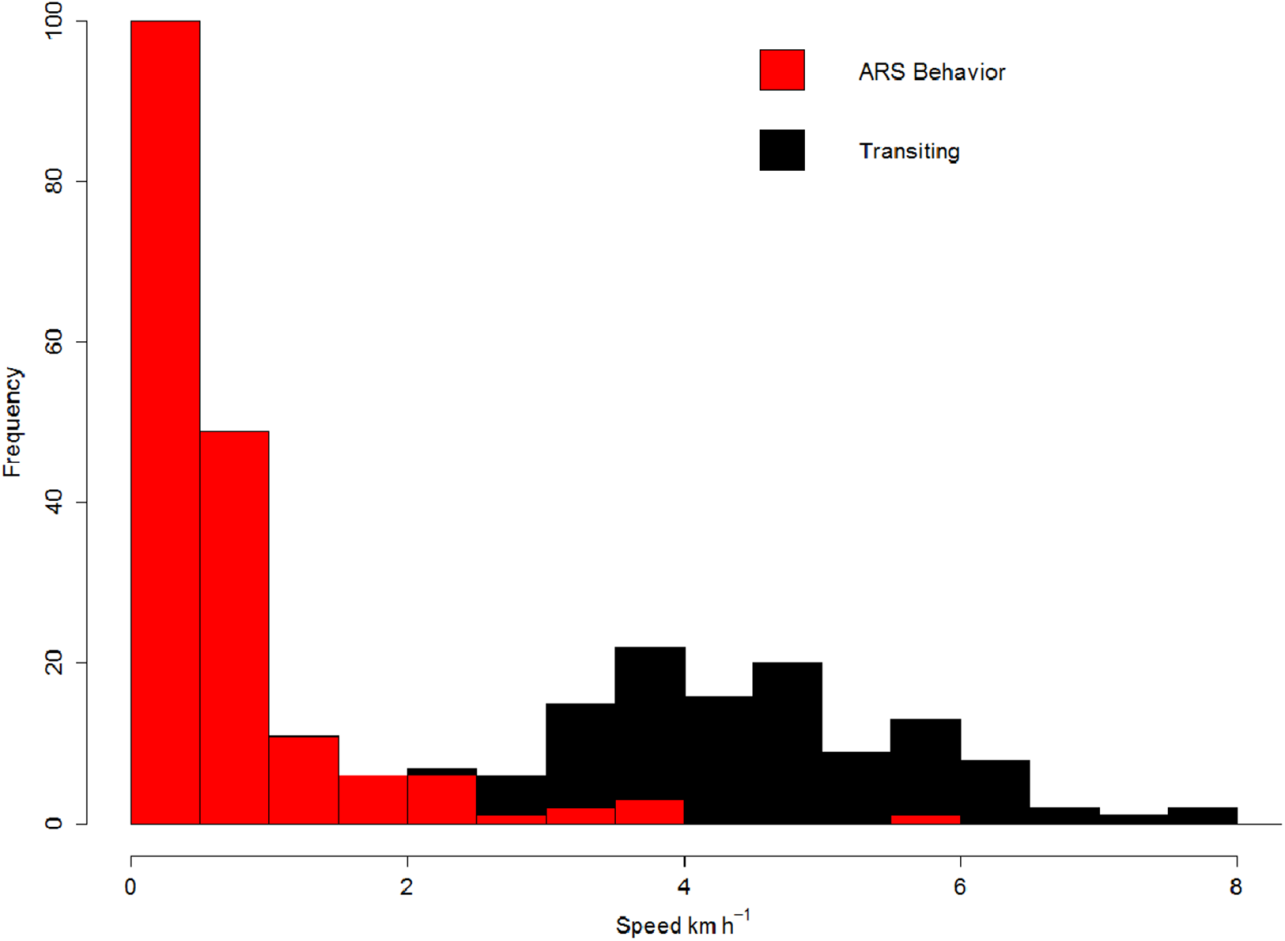
Blue whale swimming speed. Histogram of contrasting blue whale swimming speeds when engaged in different behavioral states (red: ARS, black: Transit) for all tags pooled.

Swimming speeds ranged between 0.01 and 5.7 km/h (mean 0.67 ± 0.9 km/h) during ARS behavior, while during transit these increased to 0.3–11 km/h (mean 3.87 ± 1.7 km/h) (Fig. 3; Table 2). Distance covered per day of all whales pooled indicated instrumented blue whales traveled a mean of 39 ± 27 km/day (7–85 km/day).

**Table 2:** Summary of dive data (A) for individuals #4, #5 and #7, and (B) for individual #6 along different behavioral states obtained from MK10 satellite tag data.

(A)						
Tag id	Max depth (m)		Dive duration (s)		Post dive duration (s)	
	Day	Night	Day	Night	Day	Night
4	29.2 ± 26.6 (10.5–123)	24.4 ± 22.9 (10.5–156.5)	321.3 ± 260.7 (75–1425)	278.1 ± 320.7 (75–1800)	190.3 ± 206.4 (75–1050)	195.4 ± 260.3 (75–2025)
5	25.8 ± 32.5 (12–110)	16.9 ± 7.1 (12–31.5)	258.3 ± 262.8 (75–150)	241.7 ± 149 (75–375)	358.3 ± 367 (75–1200)	808.3 ± 1192.2 (75–3375)
7	69.8 ± 41.7 (10.5–134)	25.1 ± 12.3 (10.5–65.5)	278.9 ± 164.6 (75–675)	144 ± 99.3 (75–675)	115.1 ± 205.6 (75–2175)	148.3 ± 327.8 (75–2325)

(B)						
	Max depth (m)		Dive duration (s)		Post dive duration (s)	
	Day	Night	Day	Night	Day	Night
**ARS CNP**	65.8 ± 42.1 (10.5–145.5)	21.9 ± 13.2 (11–93.5)	245.8 ± 153.5 (75–825)	192.3 ± 123.3 (75–675)	101.1 ± 98.6 (75–1200)	165.2 ± 276.1 (75–2400)
**Transit**	53.8 ± 67.6 (10.5–292)	26.5 ± 30.8 (10.5–239)	335.4 ± 286.4 (75–1350)	265.8 ± 210.1 (75–1050)	414.1 ± 684.1 (75–4350)	707.4 ± 1510.2 (75–12000)
**ARS GA**	214.9 ± 122.9 (11–336.5)	41.6 ± 33.2 (10.5–127.5)	398.7 ± 222.3 (75–975)	165.8 ± 95.5 (75–375)	142.1 ± 74.6 (75–375)	94.7 ± 42.1 (75–225)

**Figure 4:**
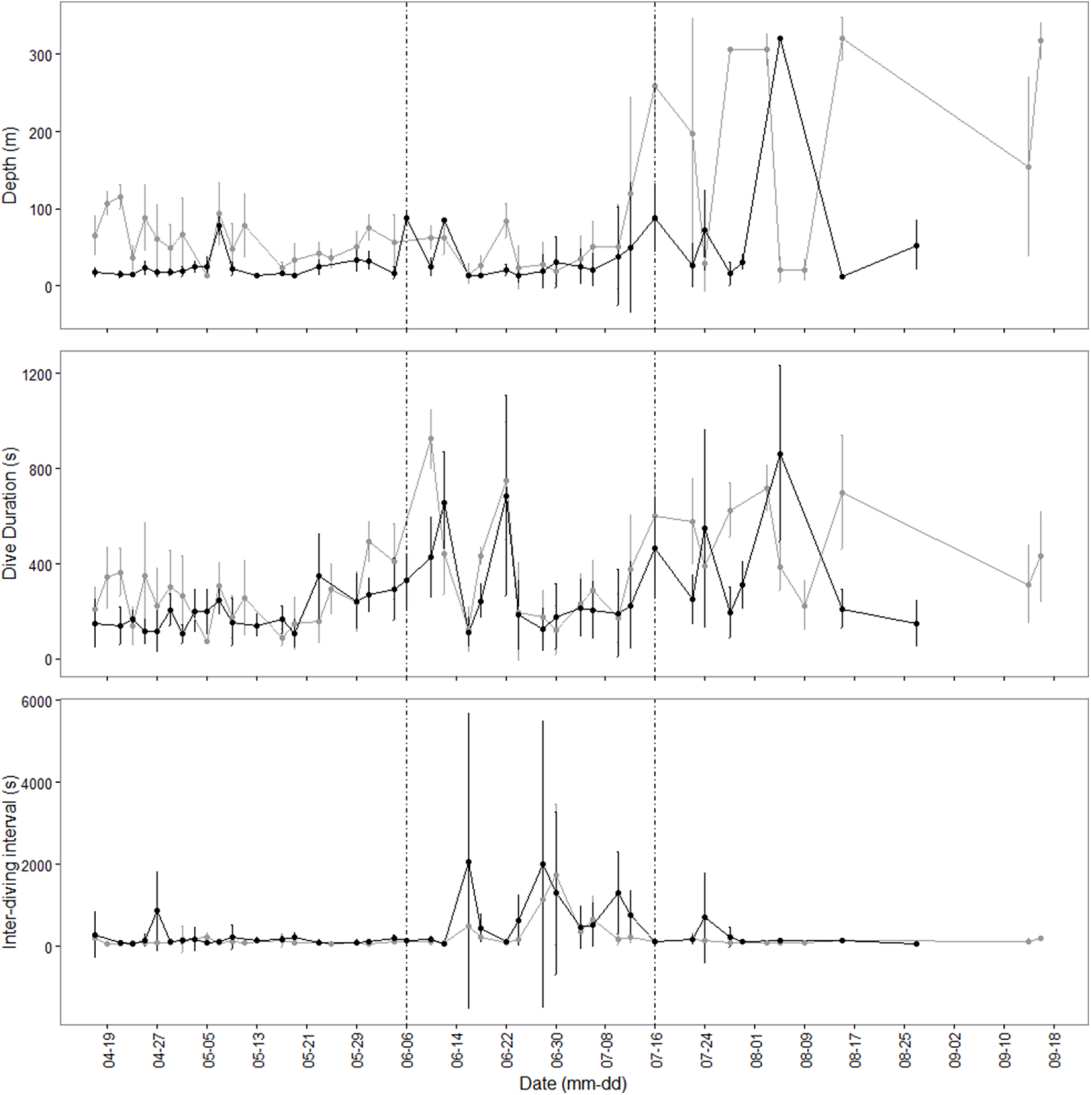
Blue whale dive profiles and durations. Dive profiles, dive durations and post-dive durations of tagged blue whale #6 over 161 days from mid-April to late-September 2015. Dashed vertical lines indicate sequential change of state in migratory behavior between ARS in CNP (left), transit along the ESP (center) and ARS in GA (right). Gray lines represent daylight and black lines night-time.

The four whales instrumented with MK10 tags provided interesting (but necessarily preliminary) data on diving depths, dive durations and inter-dive durations. At CNP, dive depths ranged from 10.5 to 156 m; during migration depths tended to be similar (regardless of bottom bathymetry) (Table 2; Fig. 4) and for the single whale that reached GA instrumented with a MK10 tag (whale #6), diving-depth dramatically increased north of 5°S, exceeding 330 m at some points, with deep dives restricted almost exclusively to daylight hours (Table 2B; Fig. 4). A diel pattern was also apparent in most dive profiles, which have a tendency to be shallower and shorter during the night and deeper and longer during daylight hours (Fig. 4). This was not only observed at CNP or GA, but also during migration, however the pattern was less pronounced and only observed in one whale (#6) which provided enough detail for different behavioral states (Table 2B).

## Discussion

Information derived from the satellite tagged blue whales in this study provides the first of its kind off the ESP. Our results provide valuable information on their migratory timings, routes and behavior on their distinctive northbound migration, particularly regarding the potentially varied plasticity in migratory patterns for individuals in this particular population. Our results also highlight the first record of two complete migratory paths between CNP and GA.

The occurrence of blue whales off GA has been documented for multiple decades. Sightings have been reported throughout the Eastern Tropical Pacific Ocean (ETP) and particularly in waters off the GA (1,000 km West of Ecuador), chiefly along the west side of the archipelago where upwelling is strong (Reilly & Thayer, 1990; Wade & Gerrodette, 1993; Merlen, 1995; Palacios, 1999). Blue whales are almost exclusively seen in that region during the austral winter and spring months (Reilly & Thayer, 1990; Palacios, 1999). This study reinforces the hypothesis that GA is a potential wintering (breeding) destination of migratory CNP blue whales (ca. 6,000 km away from the tagging study area in southern Chile) and provides the first evidence of direct migration between the two regions. Additionally, the movements performed by whale #10 when approaching the Peruvian coast prompts us to review ancillary data reported by Ramírez (1983) and Donovan (1984). The former author highlights that blue whales were observed and caught throughout the year off Paita, Peru, but peak during austral summer months (December–March) between 1961–1966 and 1975–1982. The review made by Donovan (1984) showed the peak of blue whale sighting rates (summed over the eight year period between 1976 and 1983) to occur during January and February, followed by a gradual decline until June, and increasing again from October onwards (although no effort is reported for July and August). Also, a research cruise undertaken during late 1982 (November–December) obtained 10 sightings of 14 animals within 60 nautical miles off Peru, and a further two single animals some 700 and 1,200 nm offshore. The former area coincides quite closely to the area chosen by whale #10 (Fig. 2A) to begin ARS behavior and leads us to carefully propose a working hypothesis for aiming at resolving the question posed by Donovan (1984) more than three decades ago: after leaving CNP feeding areas and reaching GA during winter-spring ESP blue whales approach Peruvian waters and continue due South along or offshore the Chilean coast to reach CNP feeding ground again.

Migratory behavior among blue whale populations worldwide appears to be largely variable. For instance, Bailey et al. (2009) used data from 128 tags deployed between 1993 and 2007 and concluded that migratory routes were primarily close to the continental margin where ARS state occurred along most of the North Pacific Coast, with occasional movements offshore. A similar pattern has been observed for tagged pygmy blue whales migrating between Australia and Indonesia in which during the northbound migration whales travelled relatively close to the western Australian coastline (about 100 km away) until reaching North West Cape, a prominent peninsula in the north-west of the state of Western Australia after which they travelled offshore about 240 km away (Double et al., 2014). This pattern of migrating close to continental margins might be regarded as an indication of feeding expectations at some specific locations along migratory routes, like the Eastern Northern Atlantic blue whale population temporarily suspending their migration at the Azores (Silva et al., 2013). By contrast, whales departing CNP travelled along widely dispersed migratory paths spread longitudinally over ca. 2,000 km and travelled up to 7,500 km, the longest blue whale migration recorded so far in the Southern Hemisphere. No ARS behavior was observed within their paths from Southern Chile to the ETP, suggesting these whales did not stop in search for potential feeding opportunities. Even when the resolution of the data would prevent us from detecting short-time periods of opportunistic feeding during migration using model 1, using finer-resolution model 2 did not provide a different outcome with data coming from whale #10.

In general, the proportion of time allocated to ARS and the size of the areas destined to this behavior by blue whales in the ESP are considerably reduced from those observed in the Eastern North Pacific, using the exact same model (Bailey et al., 2009). The ARS behavior observed at the mid-latitude feeding ground in CNP was concentrated throughout a relatively small coastal portion of roughly 300 linear km, where specific feeding patches are necessarily smaller in scale. For instance, whale #10 spent almost three months in an area of roughly 3,600 km^2^ exclusively exhibiting ARS behavior. These observations suggest that the population of blue whales summering in CNP is exploiting a predictable and highly productive habitat. In contrast, the area used for ARS in tropical waters was considerably larger (ca. 130,000 km^2^), more closely resembling the size of the areas destined for ARS in ENP and the Azores (Bailey et al., 2009; Silva et al., 2013) suggesting more sparse prey availability there. Results from model 2 in whale #10 data showed that when using finer scale resolution we can identify different areas where ARS is carried out reinforcing the idea that prey is possibly more sparse in this tropical environment.

The ARS behavior identified in the tropical wintering grounds off GA may be representative of reproductive behavior; however, preliminary analysis of diving behavior data derived from MK10 tags suggests that foraging might also be taking place. This hypothesis derives from the abrupt increase in diving depths observed from a single animal (#6) monitored extensively enough north of 5°S and the diel pattern characterized by shallower dives during nighttime. Although mixed evidence exists regarding whether blue whales feed at night (Calambokidis et al., 2007; Doniol-Valcroze et al., 2011), at least observed daytime depths reached by whale #6 matches the previously reported vertical migratory pattern of krill at GA (Brinton, 1979) and might indicate that blue whales use environmental cues or memory to adjust their diving patterns in accordance to krill distribution in that region.

Four whales tagged during 2015 off CNP exhibited the expected pattern of departing the feeding ground during mid-autumn, while one individual stayed within the Chiloe inner sea well into the austral winter when transmission ceased. During 2016, the single whale that transmitted long enough (#10), started migratory movements nearly three months later than the four whales that showed migratory behavior in 2015. Unfortunately, there is not enough information yet to infer whether this was a population response to prevailing environmental conditions during that year or an example of the individual variability in migratory behavior within the population. However, data from 2015 suggests that within-population variability occurs regarding departure time. Passive acoustic monitoring of blue whale calls suggest that blue whales depart from CNP during June/July (Buchan, Stafford & Hucke-Gaete, 2015), which is consistent with our findings. However, the extended time window where migration might occur raises the question of how profitable such prolonged delays in arriving to wintering grounds could be. Sex, age and reproductive status (Craig & Herman, 1997; Craig et al., 2003; Noad & Cato, 2007), the location of different wintering/summering grounds (Stevick et al., 2003; Kennedy et al., 2014) and shifts in oceanographic conditions (Rugh, Shelden & Schulman-Janiger, 2001; Visser et al., 2011) have been reported to determine migratory timing in baleen whales. Understanding how these factors might determine migratory patterns in ESP blue whales represent a major goal as it has been suggested that large differences in the number of blue whales arriving to CNP each year might be responsible of not yet achieving the depiction of a clear trend in CNP blue whale population size (Galletti-Vernazzani et al., 2017).

Traditionally, migration has been understood as a seasonal, persistent and predictable to-and-from movement of populations between areas where conditions are alternately favorable or unfavorable (Dingle & Drake, 2007). However, migration is based on the outcome of ecological and evolutionary processes shaped by natural selection and other evolutionary forces acting on individuals (Dingle, 2006; Dingle & Drake, 2007). Understanding how inter-individual variability observed in migration relates to variation in fitness (Bêty, Giroux & Gauthier, 2004; Hanson et al., 2008; Crossin et al., 2010; Vardanis et al., 2011) is relevant if we are attempting to understand higher (population) level repercussions of this phenomenon (Dingle, 2006).

## Conclusion

Overall, results provided here deliver important insights on blue whale habitat use in the feeding ground off CNP during austral autumn as well as providing relevant information on their migratory timing, routes and behavior on their distinctive northbound migration. The broad-scale accounts of ARS behaviors presented here for a mid-latitude feeding ground should be complemented with finer scale studies that allow the identification of potentially important feeding habitat within CNP. This is part of an undergoing work focused on movement patterns within the ESP that will aim at using shorter regular time steps in the SSM analysis, as well as the integration of environmental covariates. Over the years these studies could allow us to understand the underlying mechanisms that determine important ecological processes, such as blue whale habitat selection at different scales.

Despite the clear benefits of satellite tagging for better understanding the ecology and improving conservation of large whales, concerns have been raised with regard to the possible adverse effects of body-penetrating tags to whales, including changes in behavior and habitat use, physical injuries, prolonged health issues and decreased survival and reproductive rates (Weller, 2008). Attempts to evaluate the potential effects of tags to individual whales have not been able to demonstrate differences in the survival probability of tagged humpback whales when compared to individuals that had not been tagged (Mizroch et al., 2011; Robbins et al., 2013). However, design flaws documented in early implantable tags have resulted in higher probability of observing persistent injuries near the tag site on right, humpback, blue and gray whales (Kraus, Quinn & Slay, 2000; Norman et al., 2017; Robbins et al., 2013) and the potential for lower reproductive outputs in blue and humpback whales (Gendron et al., 2015; Norman et al., 2017). Correction of these flaws resulted in improved tag duration and minimized animal welfare problems (e.g., persistence of injuries at the tag site), but additional studies are required to assess potential effect on reproductive rates (Robbins et al., 2013). No effects of tagging were observed in the calving probabilities of Southern right whales (Best, Mate & Lagerquist, 2015) or on humpback whale reproductive success in the Southern Hemisphere (Guzman & Capella, 2017).

Predictions from the hypotheses presented here should be tested promptly in order to focus research and conservation efforts on those geographical areas most likely to provide additional evidence, possibly off Ecuadorian (during the austral late winter and spring) and Peruvian waters (during the austral summer). As the overall array of blue whale migratory routes and behaviors in the ESP are starting to become unveiled, other important feeding hotspots and breeding/calving areas still remain unknown and thus our capacity to identify and quantify threats together with implementing appropriate conservation measures, at appropriate scales remains limited and should become a priority in the coming years. Our results suggest that special care should be devoted to understanding key behavioral aspects at CNP to reduce potential ship strikes, as well as during migration. At GA there is a need to identify the potential breeding/calving site as our data indicate this area is outside the Galapagos Marine Reserve and as such is not under strict protection, but still mostly within the Exclusive Economic Zone (EEZ) of Ecuador. On this regard, we think that any successful regional conservation initiative should consider joint efforts between Chile, Peru and Ecuador to identify potential threats, evaluate effects and impacts and together propose and put into practice prospective solutions.

## Supplemental Information

10.7717/peerj.4695/supp-1 Supplemental Information 1 Argos-derived locations for blue whales. Raw data of satellite derived locations from ARGOS.

10.7717/peerj.4695/supp-2 Supplemental Information 2 BUGS code for state space models used.

## Additional Information and Declarations

### Data Availability

The following information was supplied regarding data availability: The raw data and codes used in the analysis are provided as Supplemental Files.
